# Basic Self-Disturbances Related to Reduced Anterior Cingulate Volume in Subjects at Ultra-High Risk for Psychosis

**DOI:** 10.3389/fpsyt.2019.00254

**Published:** 2019-05-10

**Authors:** Ilaria Bonoldi, Paul Allen, Luis Madeira, Stefania Tognin, Matthijs G. Bossong, Mathilda Azis, Carly Samson, Beverly Quinn, Maria Calem, Lucia Valmaggia, Gemma Modinos, James Stone, Jesus Perez, Oliver Howes, Pierluigi Politi, Matthew J. Kempton, Paolo Fusar-Poli, Philip McGuire

**Affiliations:** ^1^Department of Psychosis Studies, Institute of Psychiatry, Psychology and Neuroscience, King’s College London, London, United Kingdom; ^2^OASIS service, SLaM NHS Foundation Trust, London, United Kingdom; ^3^Department of Brain and Behavioral Sciences, University of Pavia, Pavia, Italy; ^4^Department of Psychology, University of Roehampton, London, United Kingdom; ^5^Department of Psychiatry, Icahn Medical School, Mt Sinai Hospital, New York, NY, United States; ^6^Department of Psychiatry, Brain Center Rudolf Magnus, University Medical Center Utrecht, Utrecht, Netherlands; ^7^The West London Early Intervention service, Imperial College London, London, United Kingdom; ^8^CAMEO Early Intervention Services, Cambridgeshire and Peterborough NHS Foundation Trust, Cambridge, United Kingdom; ^9^Department of Psychology, Institute of Psychiatry, Psychology and Neuroscience, King's College London, London, United Kingdom; ^10^Department of Neuroimaging, Institute of Psychiatry, Psychology and Neuroscience, King's College of London, London, United Kingdom; ^11^Department of Psychiatry, University of Cambridge, Cambridge, United Kingdom; ^12^MRC Clinical Sciences Centre (CSC), London, United Kingdom; ^13^Institute of Clinical Sciences (ICS), Faculty of Medicine, Imperial College London, London, United Kingdom; ^14^Early Psychosis: Interventions and Clinical-detection (EPIC) lab, Department of Psychosis Studies, Institute of Psychiatry, Psychology and Neuroscience, King’s College London, London, United Kingdom

**Keywords:** schizophrenia, ultra-high risk, psychosis, self-disturbances, magnetic resonance imaging, voxel-based morphometry

## Abstract

**Introduction:** Alterations of the “pre-reflective” sense of first-person perspective (e.g., of the “basic self”) are characteristic features of schizophrenic spectrum disorders and are significantly present in the prodromal phase of psychosis and in subjects at ultra-high risk for psychosis (UHR). Studies in healthy controls suggest that neurobiological substrate of the basic self involves cortical midline structures, such as the anterior and posterior cingulate cortices. Neuroimaging studies have identified neuroanatomical cortical midline structure abnormalities in schizophrenic spectrum disorders.

**Objectives:** i) To compare basic self-disturbances levels in UHR subjects and controls and ii) to assess the relationship between basic self-disturbances and alterations in cortical midline structures volume in UHR subjects.

**Methods:** Thirty-one UHR subjects (27 antipsychotic-naïve) and 16 healthy controls were assessed using the 57-item semistructured Examination of Anomalous Self-Experiences (EASE) interview. All subjects were scanned using magnetic resonance imaging (MRI) at 3 T, and gray matter volume was measured in *a priori* defined regions of interest (ROIs) in the cortical midline structures.

**Results:** EASE scores were much higher in UHR subjects than controls (*p* < 0.001). The UHR group had smaller anterior cingulate volume than controls (*p* = 0.037). There were no structural brain imaging alterations between UHR individuals with or without self-disturbances. Within the UHR sample, the subgroup with higher EASE scores had smaller anterior cingulate volumes than UHR subjects with lower EASE scores and controls (*p* = 0.018). In the total sample, anterior cingulate volume was inversely correlated with the EASE score (*R* = 0.52, *p* < 0.016).

**Conclusions:** Basic self-disturbances in UHR subjects appear to be related to reductions in anterior cingulate volume.

## Introduction

The psychopathological construct of basic self-disturbances is based on the pre-conscious sense of self, termed “*basic self*,” as opposed to conscious, reflective, and more elaborated levels of self-awareness (1, 2). This pre-reflective, implicit sense of self indexes a first-person perspective on the world (3). Abnormalities in basic self may result in alterations of the subjective sense of being a vital subject at the center of one’s own experience (4). There is emerging evidence suggesting that *basic self-disturbances* are a key feature of the schizophrenic spectrum disorders (2) and that the presence of *basic self-disturbances* may distinguish schizophrenia from affective psychosis (5, 6) and other psychiatric disorders (6–9). *Basic self-disturbances* are nonpsychotic abnormalities of experience that could evolve in frank psychotic symptoms. For example, an altered sense of “ownership” of one’s own experience can lead to thoughts being experienced as alien, eventually resulting in psychotic phenomena such as believing that one’s thoughts come from an external source (thought insertion).

*Basic self-disturbances* have been reported in samples at genetic high risk for schizophrenia (10), at ultra-high risk (UHR) for psychosis (11), and in the prodromal phase of schizophrenia (12, 13). The UHR construct identifies subjects with an increased risk of developing psychotic disorders [20% at 2 years, see Table 4 in Ref. (14)] (15) but not of other nonpsychotic disorders (16). The vast majority (73%) of UHR subjects who develop psychosis will develop a schizophrenia spectrum psychosis (17). The increased risk that is observed in these individuals is mostly due to the accumulation of several risk factors for psychosis (18, 19) during the sampling and the recruitment of these individuals (20, 21). Recent evidence suggests that basic self-disturbances in UHR subjects are related to the risk of subsequently developing psychosis (particularly schizophrenic spectrum) (11).

Despite the large array of structural neuroimaging investigations in UHR individuals (22–26), the neurobiological substrate of *basic self-disturbances* is unknown, but some authors have suggested (27) that in healthy individuals, cortical midline structures, particularly anterior cingulate cortex (ACC), posterior cingulate cortex (PCC), and medial prefrontal cortex, represent the neural basis of the basic self (28).

In fact, a variety of brain regions are involved in self-referential processing requiring an active reflection on self (e.g., recognizing personality traits as belonging to self or others) (29). However, cortical midline structures are robustly activated in all self-referential tasks, regardless of the sensory mode within which the self-stimuli were presented (30). Therefore, they are postulated to be the basis of the pre-reflective (basic) self, which precedes and allows any more elaborated level of self-awareness.

A meta-analysis of functional imaging studies has identified three clusters within cortical midline structures (30), constantly recruited in self-related tasks in healthy volunteers, independent of the sensory modalities: 1) pre- and sub-genual ACC/ventromedial prefrontal cortex, 2) supra-genual ACC/dorsomedial prefrontal cortex, and 3) PCC. Collectively, these areas are implicated in the evaluation and representation (medial prefrontal cortex), monitoring (ACC), and integration of self-referential stimuli (PCC).

Both structural and functional neuroimaging studies of UHR subjects have reported alterations in cortical midline structures. Thus, magnetic resonance imaging (MRI) studies have described reductions in gray matter volume in UHR subjects in the ACC (31, 32), PCC and precuneus (33, 34), and medial frontal gyrus (31, 32). Functional MRI studies have reported alterations in activation in these regions in UHR subjects across a range of cognitive and emotional tasks (35–40). Furthermore, within UHR samples, alterations in the medial prefrontal cortex (31, 32, 41), ACC and PCC, and the precuneus (33) have been associated with the subsequent transition to psychosis. However, the extent to which alterations in cortical midline structure regions in UHR subjects relate to *basic self-disturbances* has not yet been investigated. Investigating these features can be important to improve the detection and the prediction of outcomes in UHR subjects at an individual level.

The present study was designed to address this issue. We used magnetic resonance imaging (MRI) to measure the volume of cortical midline structures regions in UHR subjects and healthy controls, and used the Examination of Anomalous Self-Experience (EASE) to assess *basic self-disturbances* in these subjects. We tested the following hypotheses: i) UHR subjects have higher levels of *basic self-disturbances* than controls; ii) UHR subjects have less gray matter volume than controls in the ACC, PCC, and medial prefrontal cortex; iii) within UHR subjects, the severity of *basic self-disturbances* is related to reductions in the volume of these regions.

## Materials and Methods

### Subjects

Thirty-one participants meeting Comprehensive Assessment of the At Risk Mental State (CAARMS) 12/2006 (42) criteria for the At Risk Mental State (ARMS) were recruited from “Outreach and Support in South London, OASIS” (https://www.meandmymind.nhs.uk) in South London and The Maudsley (43), “The West London Early Intervention service” (www.wlmht.nhs.uk/services/e/early_intervention_hf.html) in West London, and the “Cambridgeshire and Peterborough early intervention services, CAMEO” in Cambridge (http://www.cameo.nhs.uk), between November 2011 and March 2014. The neuroimaging study protocol was approved by the National Research Ethics Service Committee of London—Camberwell St Giles, United Kingdom, and all participants gave written informed consent. The UHR status was based on clinical assessment using the CAARMS (44) and a consensus meeting with the clinical team. An individual meets inclusion criteria for the ARMS if they present one or more of the following: 1) “attenuated” psychotic symptoms (APS); 2) frank psychotic symptoms that last less than 7 days and resolve spontaneously without treatment, i.e., brief limited intermittent psychotic symptoms (BLIPS); 3) a recent decline in function together with either schizotypal personality disorder or a first-degree relative with psychosis, i.e., genetic risk and functional deterioration (GRD). Four of the UHR participants were taking low-dose antipsychotic medications, while 27 were antipsychotic-naïve.

Healthy controls (HC) participants (*n* = 16) were recruited *via* advertisement in the local media. All subjects lived in the same geographical areas as clinical subjects; were matched for age, ethnicity, and premorbid IQ; and had an absence of personal or family history of psychiatric illness.

Participants for both groups were excluded if there was a history of neurological disorder or they met *Diagnostic and Statistical Manual of Mental Disorders, Fourth Edition (DSM-IV)* criteria for substance abuse.

### Clinical Assessment

#### Assessment of Ultra-High Risk Symptoms

Severity of UHR symptoms was assessed using the following instruments: the Comprehensive Assessment of the At Risk Mental State (CAARMS 12/2006) (44), the Positive and Negative Symptom Scale (PANSS) (45), the Hamilton Depression Rating Scale (HAM-D) (46), and the Hamilton Anxiety Rating Scale (HAM-A) (47). Level of functioning was assessed using the Social and Occupation Functioning Assessment Scale (SOFAS) (48). Premorbid estimated IQ was assessed by using the National Adult Reading Test (NART) (49), and current IQ was assessed with the shortened version of the Wechsler Adult Intelligence Scale (WAIS-III) (50).

#### Assessment of Basic Self-Disturbances

Basic self-disturbances were investigated in both UHR and HC with the Examination of Anomalous Self-Experience (EASE) (51) by two psychiatrists (IB and LM), who attended a certified EASE training in Copenhagen. The two psychiatrists assessed a subset of the present sample independently, to standardize the procedure. The EASE is a semistructured interview that has shown a good to excellent internal consistency (Cronbach’s α above 0.87) and an overall inter-rater correlation coefficient above 0.80 (52). It systematically explores the nonpsychotic abnormalities of experience articulating around the basic disturbance of self-awareness. The 57 items are grouped into five non-mutually exclusive domains: 1) cognition and stream of consciousness, 2) self-awareness and presence, 3) bodily experience, 4) demarcation/transitivism, and 5) existential reorientation. These items are then rated either dichotomously (present = 1 or absent = 0) (53) or continuously on a five-point severity and frequency scale (11). For the purpose of this study, the interview was rated continuously, and item subtypes were included in the scores.

### Magnetic Resonance Imaging Scanning

For all participants, images were acquired at the Centre for Neuroimaging Sciences, Institute of Psychiatry, King’s College London on a 3-T Signa HDx (General Electric, Milwaukee, WI). T1-weigthed scans were obtained using a volumetric three-dimensional Spoiled Gradient Recalled sequence (slice thickness = 1.2 mm, TE = 2.8 ms, TR = 6.98 ms, TI = 400 ms, flip angle = 11°, matrix = 256 × 256) producing 196 sagittal slices with an in-plane resolution of 1.0 × 1.0 mm.

### Data Analysis

#### Clinical Measures

Differences in demographic and clinical variables between groups were examined using independent samples *t* tests for parametric and continuous data and a χ^2^ test for categorical data using SPSS (version 19.0 for Mac; Statistical Package for the Social Sciences (SPSS) Inc., Chicago, Illinois). Mann–Whitney *U* test was used to assess differences in EASE scores between HC and UHR as EASE scores were not normally distributed.

#### Image Analysis

Between-groups differences in gray matter volume were assessed using voxel-based morphometry (VBM), as implemented in Statistical Parametric Mapping (SPM8) software (http://www.fil.ion.ucl.ac.uk/spm), running under MATLAB 8.2 (The MathWorks, Inc, Natick, MA). T1-weighted volumetric images were preprocessed using the DARTEL (54) SPM8 toolbox. This technique maximizes accuracy and sensitivity, as it creates a study-specific template and the segmentation of each individual image (55). VBM preprocessing was conducted as follows: 1) visually checking for scanner artifacts and gross anatomical abnormalities for each subject, 2) setting the image origin to the anterior commissure, 3) using the DARTEL toolbox to produce a high-dimensional normalization protocol, 4) checking for homogeneity across the sample, and 5) using standard smoothing (i.e., 8 mm). We also included a “modulation step” in the normalization to preserve the information about the absolute gray matter values (56). After this preprocessing, smoothed, modulated, normalized data were obtained and used for the statistical analysis.

We examined three *a priori* regions of interest (ROIs) in the ACC, PCC, and medial frontal gyrus. Using the SimpleROIBuilder toolbox (http://www.fil.ion.ucl.ac.uk/spm/ext/), we created a single mask that included the three preselected ROIs. Within the mask, statistical inferences were made at *p* < 0.05 and family-wise error (FWE) rate correction, using an analysis of covariance (ANCOVA) design to identify significant differences in gray matter volume across UHR and HC, with age, gender, years of education, and total intracranial volume as covariates of no interest.

These ROIs were chosen, as they were the anatomical areas postulated by metanalytical literature to be the neurobiological underpinning of basic self (30).

For the correlation analysis, we used independent values, extracting the gray matter volume parameters from the peak coordinates of the three clusters 1) pre- and sub-genual ACC/ventromedial prefrontal cortex, 2) supra-genual ACC/dorsomedial prefrontal cortex, and 3) PCC derived from the metanalytical independent study (30), not to violate the assumption of independence (57).

#### Correlations Between Gray Matter Volume and Examination of Anomalous Self-Experiences Scores

To test our hypothesis that EASE scores are directly related to alterations in cortical midline structure volume, EASE scores were regressed onto the gray matter volume parameters in the peak cluster coordinates indicated in previous meta-analyses (30), after the coordinates have been converted from Talairach to Montreal Neurological Institute (MNI). Individual gray matter volume parameters from each of these peak coordinates within each cluster were extracted. Spearman’s correlation was performed in SPSS between these values and EASE scores:

Cluster 1: Ventromedial prefrontal/pre- and sub-genual ACC (*x* = −1.29, *y* = 54.1, *z* = −1.57)

Cluster 2: Dorsomedial prefrontal/supra-genual ACC (*x* = 0.38, *y* = 16.72, *z* = 48.56)

Cluster 3: PCC/precuneus (*x* = −1.84, *y* = −60.39, *z* = 36.38)

Statistical inferences were made at *p* < 0.05 FWE corrected. A Bonferroni correction for multiple testing was also applied (*p* < 0.05/3 = 0.016), and sensitivity analyses were repeated in the subsample that was drug-naïve.

#### Gray Matter Differences Between Healthy Controls and UHR With High and Low Level of Self Disorders

To examine whether a high level of *basic self-disturbances* within the UHR group was associated with altered gray matter volume in cortical midline structures, one-way analysis of variance and *post hoc* test were performed to test the effect of group (UHR-High-EASE vs. UHR-low-EASE) on gray matter volume in each of the ROIs. Statistical threshold was set at *p* < 0.05, Bonferroni correction.

## Results

### Sample Characteristics

Almost all UHR participants (*n* = 28) met ARMS criteria for APS alone, two met criteria for BLIPS alone, and one for GRD + APS. The two groups did not statistically differ for age, gender, or ethnicity, but HC had spent significantly more years in education, as compared to UHR subjects (*p* = 0.013, mean difference = 2.53 years) and significantly more of them were employed as compared to UHR individuals. As expected, UHR subjects had reduced levels of functioning relative to HC and higher levels of anxiety and depression. All UHR individuals were drug-naïve, with the exception of four individuals. The antipsychotics taken by four participants at the time of the study were as follows: quetiapine 50 mg OD (two participants), olanzapine 10 mg Once Daily (OD), and olanzapine 5 mg OD. Three of them belonged to the UHR with high self-disturbances, with one belonging to the UHR with low self-disturbance (the one taking olanzapine 5 mg). See **Table 1** for full statistical details. Over 2 years of follow-up, seven individuals developed a psychotic disorder (23%).

**Table 1:** Clinical and sociodemographic characteristics of the sample.

Categorical variables		*HC (%)*	*UHR (%)*	*χ^2^ (DOF)*	*p*
**Gender**				3.0 (1)	0.81
	*Male*	5 (31.3)	18 (58.1)		
	*Female*	11 (68.8)	13 (40.9)		
**Ethnicity**				8.0 (3)	0.220
	*White*	12 (75)	17 (54.8)		
	*Black*	1 (6.3)	11 (35.5)		
	*Asian*	2 (12.5)	0 (0)		
	*Other*	1 (6.3)	2 (6.5)		
**Employment**				5 (1)	0.025*
	*Unemployed*	1 (6.3)	11 (35.5)		
	*Employed or student*	15 (93.8)	19 (61.3)		
**UHR subgroup**					
	*APS*	n.a.	29 (90.3)		n.a.
	*BLIPS*	n.a.	2 (6.5)		n.a.
	*GRD*	n.a.	1 (3.2)		n.a.
Continuous variables		HC (SD)	UHR (SD)	F (DOF)	p
**Age**		24.9 (3.3)	23.3 (4.3)	2.3 (45)	0.204
**Years of education**		16.2 (3.21)	12.8 (2.3)	2 (44)	0.01*
**NART**	tot 50	28.7 (7.6)	27.9	0.04 (42)	0.736
**EASE**					
	*Overall*	6.5 (8.2)	117.3 (68.6)	15.0 (45)	<0.001*
	*Cognition and stream of consciousness*	2.7 (5)	42.4 (23.2)	33.6 (45)	<0.001*
	*Self-awareness and presence*	1.8 (3.7)	49.5 (27.3)	15.6 (45)	<0.001*
	*Bodily experiences*	0.19 (0.75)	11.8 (12.8)	12.9 (45)	<0.001*
	*Demarcation/transitivism*	0 (0)	3.4 (5.3)	9.0 (45)	<0.001*
	*Existential reorientation*	1.50 (3.8)	10.9 (10)	20.6 (45)	<0.001*
SOFAS		92.4 (3.3)	60.0	14.4 (41)	<0.001
HAM-A		1.5 (1.7)	16.8 (10.7)	26.3 (36)	<0.001
HAM-D		0.2 (0.6)	15.8 (10)	22.4 (35)	<0.001
CAARMS	*Total symptoms*	n.a.	39.6 (24.0)	n.a.	n.a.
	*Total positive symptoms*	n.a.	11.39 (6.1)	n.a.	n.a.
	*Total negative symptoms*	n.a.	7.9 (6.1)	n.a.	n.a.
	*Total cognitive symptoms*	n.a.	3.4	n.a.	n.a.
PANSS	*Total symptoms*	n.a.	12.6	n.a.	n.a.

*Significant differences at p < 0.05 corrected for multiple comparisons. HC, healthy controls; UHR, ultra-high risk for psychosis; APS, attenuated psychotic symptoms; BLIPS, brief, limited intermittent psychotic symptom; GRD; genetic risk + functional deterioration; NART, National Adult Reading Test; EASE, Examination of Anomalous Self-Experience; SOFAS, Social and Occupation Functioning Assessment Scale; HAM-A, Hamilton Anxiety scale; HAM-D, Hamilton Depression Scale; PANSS, Positive and Negative Symptoms Scale; DOF, degrees of freedom; SD, standard deviation; n.a., not available; tot, total.

### Assessment of Self-Disorders

The UHR group showed greater levels of *basic self-disturbances* compared to controls [overall continuous EASE score UHR 117.32 (68.6) vs. HC 6.5(8.2), Mann–Whitney *U* test *p* < 0.00] in all five EASE domains. The interview took an average of 134 min (SD = 40) in UHR and 58 min (SD = 10) in HC to complete, usually over one or two sessions. No subject failed to complete the interview. See **Table 1** for full statistical details.

When the two UHR groups (high levels of self-disturbances vs. low levels of self-disturbances) were compared in relation to HAM-A, HAM-D, Global Assessment of Functioning (GAF) disability, TOT PANSS, total (TOT) CAARMS, and relative four positive symptoms subscales, only differences between HAM-A (24.3 vs. 9.78, *p* < 0.001), HAM-D (24.4 vs. 8.62, *p* < 0.001), and Global Assessment of Functioning (GAF) disability (56 vs. 63, *p* < 0.05) were significant.

In order to investigate the effect of self-disturbances on cortical midline structure gray matter volume, the UHR group was divided into subgroups according to the median, as the scores were not normally distributed, resulting in subjects with higher EASE scores (≥median of EASE scores = 108, *n* = 15) and lower EASE scores (<median of EASE scores, *n* = 16). We then compared cortical midline structures volume in these subgroups and HC.

#### Between-Group Differences in Pre-Selected Regions of Interest (ROIs)

**Figure 1:**
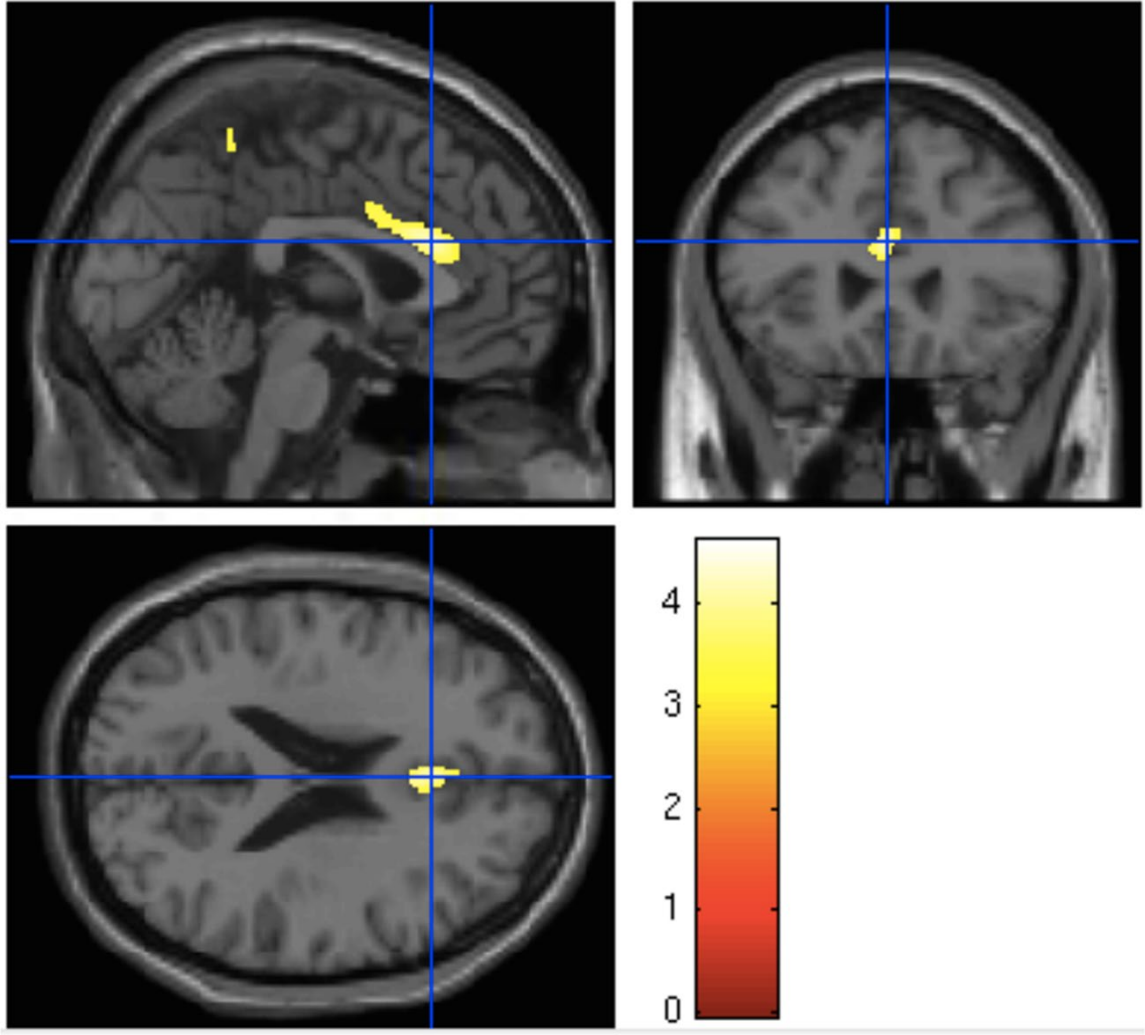
Significant reduction of gray matter volume in the anterior cingulate gyrus in ultra-high risk for psychosis subjects relative to controls [*p* = 0.037; family-wise error (FWE)].

The UHR group has reduced gray matter volume relative to the control group in the ROI centered on the dorsal ACC (MNI coordinates *x* = 0, *y* = 26, and *z* = 22; *p* = 0.037 (FWE); *z* = 3.76; and cluster size = 332 voxels) (**Figure 1**). There were no significant group differences in the superior medial frontal or posterior cingulate ROIs.

### Gray Matter Differences Between Healthy Controls and UHR With High EASE Scores and UHR With Low EASE Scores

**Figure 2:**
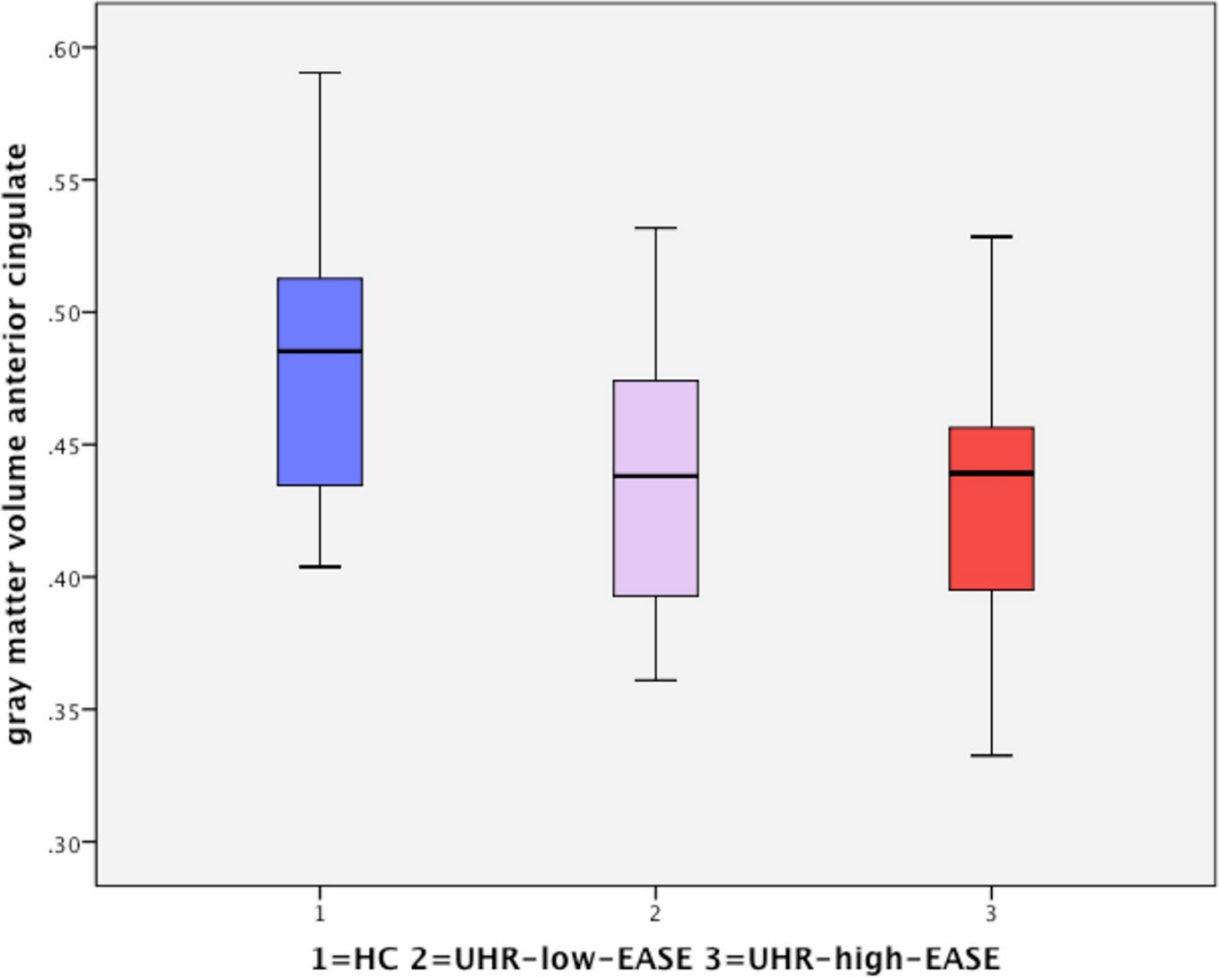
Boxplot showing gray matter volume in the anterior cingulate in the three groups: HC (healthy controls), UHR-low-Examination of Anomalous Self-Experiences (EASE), and UHR-high-EASE. Values on the *y*-axis refer to mm^3^ per voxel.

**Figure 3:**
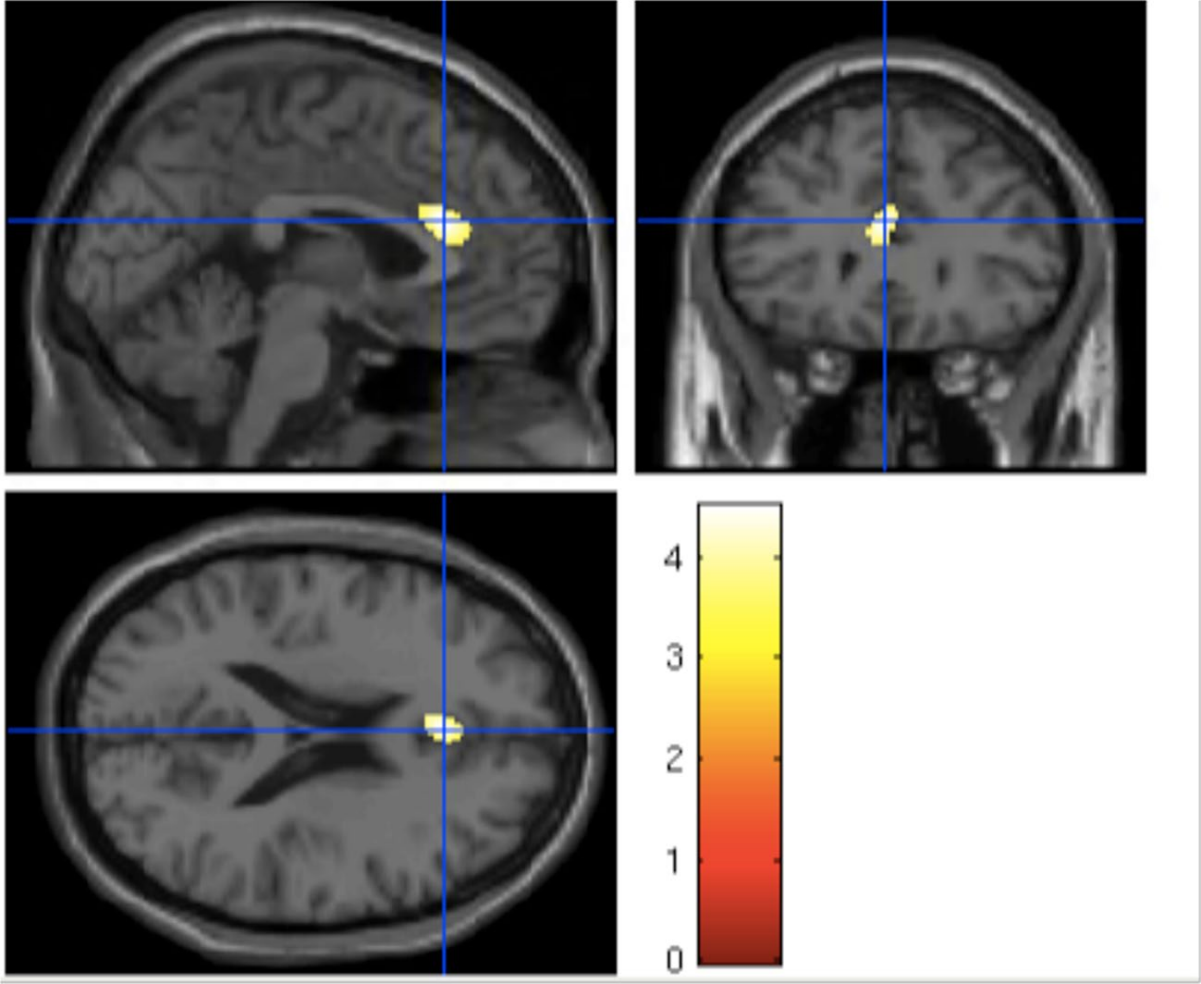
Significant reduction in the anterior cingulate volume in UHR subjects with high EASE scores compared to HC.

One-way analysis of variance found a significant effect of group on gray matter volume (*p* = 0.04) in the dorsal anterior cingulate cortex (dorsal ACC). *Post hoc* *t* tests showed significant differences in the dorsal anterior cingulate only between HC and the UHR-high-EASE subgroup (*p* = 0.018), but not for HC vs. UHR-low-EASE (*p* = 0.052) or UHR-high-EASE vs. UHR-low-EASE (*p* = 0.65). See **Figures 2** and **3**.

### Correlations Between Self-Disorders and Gray Matter Volume in Cortical Midline Structures

**Figure 4:**
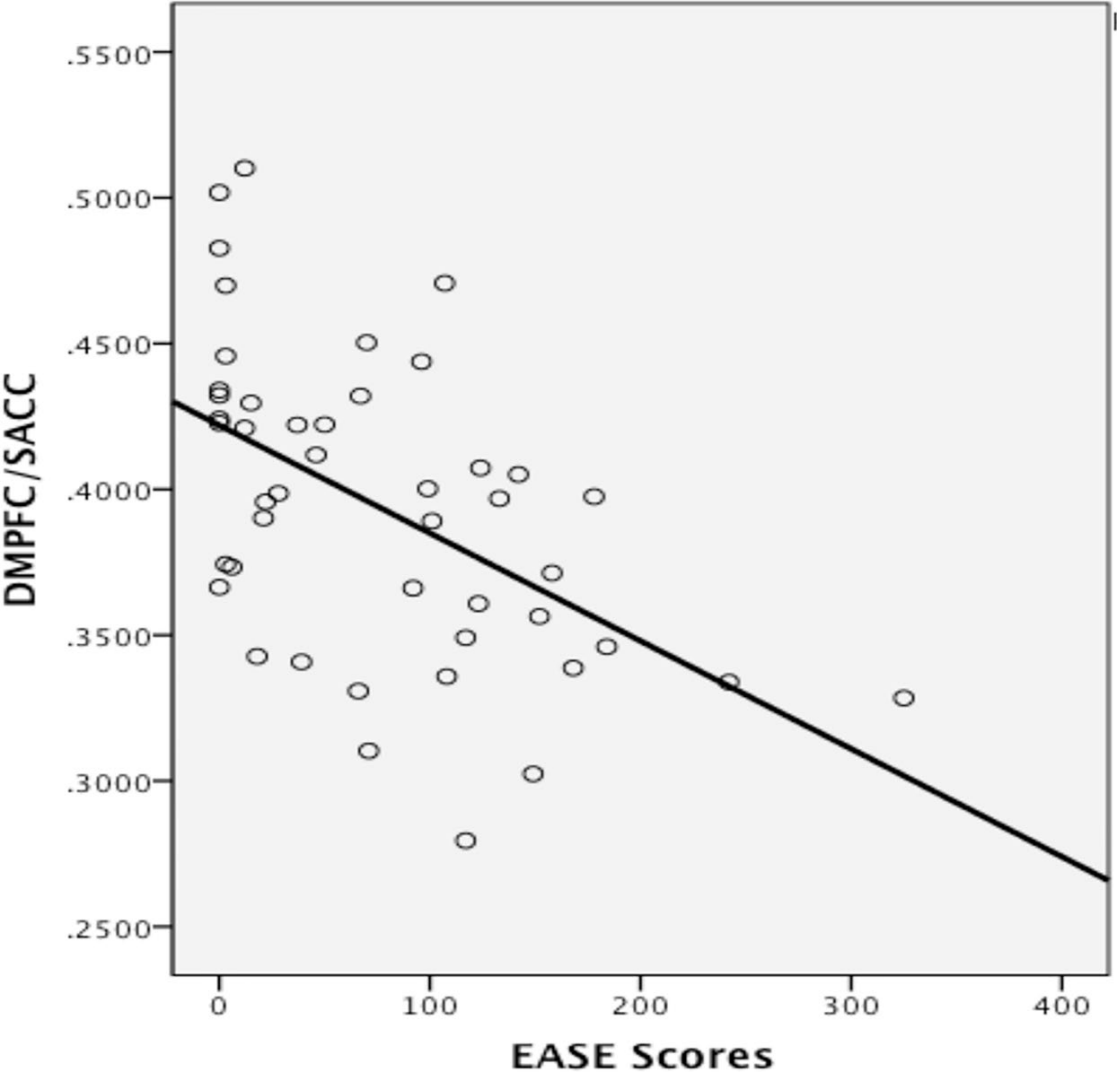
Correlation between gray matter volume in the dorsomedial prefrontal (DMPFC)/supra-genual anterior cingulate cortex (ACC) cluster and levels of self-disturbances measured with the EASE. Values on the *y*-axis refer to mm^3^ per voxel.

Spearman’s rho correlation between continuous EASE scores and gray matter volume in the ventromedial prefrontal/pre- and sub-genual anterior cingulate cluster and in the dorsomedial prefrontal/supra-genual anterior cingulate cluster were significant (*p* = 0.021, −0.33 *r* ^2^ = 0.141, and *p* < 0.001, −0.553 *r* ^2^ = 0.24, respectively, see **Figure 4**). Outliers were detected *via* Cook’s distance test. Four outliers were present in the correlation with the ventromedial prefrontal/pre- and sub-genual anterior cingulate cluster and one in the correlation with the dorsomedial prefrontal/supra-genual anterior cingulate cluster. Only the negative correlation between EASE score and the latter remained significant after the outlier had been removed (*r* ^2^ = 0.269, *p* < 0.001). Likewise, after Bonferroni correction for multiple comparisons, only the correlation in this cluster remained significant (*p* < 0.016).

No significant correlation was found between EASE scores and the volume of the posterior cingulate/precuneus cluster.

No correlations were found between CAARMS (total, positive, negative, cognitive, and general symptoms scores) or PANSS (total and positive symptoms) scores and the three gray matter volume clusters in the cortical midline structures.

HAM-A (*p* < 0.001), HAM-D (*p* < 0.05), and SOFAS (*p* < 0.05) correlate with the third cluster dorsomedial prefrontal/supra-genual ACC only.

Excluding those four participants who had received an antipsychotic medication, the correlation between self-disturbances and gray matter volume remains significant for the ventromedial prefrontal/pre- and sub-genual ACC and dorsomedial prefrontal/supra-genual ACC.

## Discussion

This is the first study to directly examine the association between basic self-disturbances and gray matter volume in a population of UHR subjects. Our first prediction was that UHR participants would have higher EASE scores than HC. This hypothesis was confirmed. These results replicate previous findings in two different UHR samples (11, 58), further confirming literature suggestions (10, 12, 13) that abnormalities of the basic self are nonpsychotic alterations of self-awareness that precede the onset of full blown psychosis and are core features of vulnerability to psychosis.

Our second prediction was that UHR subjects would show gray matter volume reductions relative to HC in the cortical midline structures regions that are implicated in self-referential processing. We found that UHR subjects had lower gray matter volume than HC in the ACC, one of the cortical midline structures. Previous MRI studies have reported structural alterations of ACC in UHR populations (31, 32, 34), but these have not been specifically related to basic self-disorders. A secondary analysis indicated that this reduction in ACC volume was influenced by the subgroup of UHR subjects with relatively higher level of self-disturbances, as measured with the EASE: ACC volume in UHR subjects with lower EASE scores was lower, but not significantly different to that in HC. Finally, a correlational analysis involving all the participants (UHR plus HC) revealed that ACC volume was inversely related to EASE score: the higher the level of self-disturbances, the lower the gray matter volume in the ACC.

Our main findings involved the dorsal part of the ACC, an area that has been implicated in mediating attention, cognitive/attentional control, conflict monitoring, response inhibition, and self-reflection (59–62). It also plays a role in the integration of rewarding environmental cues and behavioral responses, *via* its widespread projections to affective, cognitive, and motor cortices (63). The motivation of behavior in relation to reward relies on the attribution of salience to environmental stimuli. Salience models of psychosis propose that aberrant attribution of salience to irrelevant environmental stimuli underlies the development of positive psychotic symptoms (64). It has previously been suggested that dysfunctional salience processing may also contribute to emergence of basic self-disturbances (65, 66): the capacity to compare predicted and incoming stimuli would be altered, resulting in a violation of expectation. If such a prediction error does not fit the knowledge based on previous experience, a new inference occurs (67). These prediction errors make an event attention grabbing, i.e., more salient, which could result in basic self-disturbances such as a loss of “common sense” (i.e., a disruption of a person’s “grasp” on the conceptual or perceptual field of awareness, loss of the implicit “grip” of the “rules of the game,” of the ability to see things in the proper perspective), hyper-reflexivity (a tendency to constantly monitor one’s own experience, normally tacit in the “background”) (2, 68), and diminished self-presence (lack of vital contact, diminished sense of existence as a subject of awareness) (66, 69).

Sense of agency (e.g., while performing an action) would derive from the comparison of predicted (expected) and actual sensation: concordance signifies that the movement is one’s own, while discrepancy suggests that the movement is externally generated. A similar process is thought to underlie sense of agency of mental content (cognitive–affective agency). The dorsal ACC and prefrontal cortex, *via* their interactions with motivational (ventral striatum) and limbic (amygdala) areas, are thought to play an important role in the sense of being a “cognitive-affective agent” (e.g., the agent and owner of mental content and affect) (70).

Different neurocognitive models of psychosis propose that symptoms such as auditory hallucinations and delusions of control may derive from misattribution of self-generated actions as externally generated as a consequence of a dysfunctional self-monitoring mechanism (71, 72).

In the motor domain, prediction of the sensory consequences of planned actions allows discrimination of self- and non-self-elicited sensation (73). Shergill et al. recently demonstrated that schizophrenia patients seem unable to predict the sensory consequences of their own actions (74). According to the conflict-monitoring model (75), an evaluative/regulative loop mediated by dorsal ACC (evaluative component) and PFC (regulatory component) would allow a self/nonself distinction between reafferent signals resulting from one’s own cognitive control efforts (self) and exafferent signals about the level of conflict resulting from environmental sources (nonself) (70). Alterations in the dorsal ACC could therefore impair the self/nonself distinction and underlie basic self-disturbances such as loss of sense of agency and ownership of mental content (thoughts felt as alien, thought interference and insertion) and alteration of the first-person perspective, eventually resulting in psychotic passivity phenomena.

The results of the current study support the role of the ACC in the pathogeneses of basic self-disturbances.

A previous study in a UHR sample demonstrated that structural changes in the ACC appear before the onset of frank psychosis, can distinguish between UHR who will subsequently develop psychosis compared to those who will not, and seem relatively specific to UHR individuals who develop schizophrenia spectrum disorders, as opposed to affective psychoses (31). Volumetric changes in the ACC are also among the most robust neuroanatomical alterations in patients with established schizophrenia (76). This is in line with the notion that basic self-disturbances tend to segregate in the schizophrenic spectrum (6, 9) as opposed to affective psychosis (5) or borderline personality disorder (77).

The EASE interview targets nonpsychotic abnormalities of conscious experience that are not included in conventional psychopathological assessments of UHR symptoms, such as the CAARMS (44). Incorporating the EASE into the routine clinical assessment of UHR subjects may facilitate risk stratification and the provision of individualized interventions.

Our study has several limitations. The first one is the lack of follow-up neuroimaging data. Follow-up scan could inform on the longitudinal trajectory of the neuroanatomical alterations detected in our UHR subjects, while only functional and clinical outcome could shed light on the diagnostic and prognostic validity of our findings. Diagnostic and prognostic information can in turn support risk stratification and personalized focused interventions in early psychosis (78, 79).

The second limitation is the small sample size, which limits the validity of our results and the possibility to generalize them to the broader UHR population. Moreover, due to small numbers (two BLIPS and one GRD), we have been unable to stratify our findings across different UHR subgroups (APS, BLIPS, and GRD). These three groups have been found to be heterogeneous in terms of psychotic risk, with BLIPS having a significant higher risk to develop psychosis as compared to APS and GRD, and GRD not showing an increased risk of developing psychosis in the short term (4 years) (14, 80, 81). In particular, the BLIPS group, which resembles the Acute and Transient Psychotic Disorder group defined by the International Classification of Diseases, tenth revision (ICD-10) (81), is characterized by specific unmet needs and poor longer-term outcomes, beyond the heightened risk of developing psychosis (82, 83).

This heterogeneity could confound both clinical and neuroanatomical findings. This can also be the cause for the lack of neuroanatomical differences between the UHR individuals with and without self-disturbances. It is thus possible that to detect these neuroanatomical effects, a larger sample would be needed. Third, in our study, we could not control for affective comorbidities, as in our sample, EASE scores positively correlated with levels of anxiety and depression. This is a potential limitation, as comorbid depression and anxiety disorders significantly contributed to gray matter volume reductions of the ACC in people at UHR of psychosis in a previous study (84).

Finally, these preliminary results need to be replicated in different larger samples and in longitudinal neuroimaging study designs.

## Conclusions

The data from the present study suggest that high scores on the EASE in UHR subjects, which reflect subjective disorders of the self, are related to reductions in the volume of the ACC. These findings represent a first step forward toward the integration of subjective experiences of self and neurobiological alterations in the early phase of psychosis. Further studies integrating phenomenological, neurocognitive, and neurobiological aspects of basic self-disturbances are warranted to improve our understanding of the role of self-disorders in vulnerability to psychosis.

## Ethics Statement

The study protocol was approved by the National Research Ethics Service Committee of London-Camberwell St Giles, United Kingdom, and all participants gave written informed consent.

## Author Contributions

IB conducted the study under the supervision of PA and drafted the version of the manuscript. LM contributed the administration of the EASE questionnaire. ST helped analyzing VBM data. MB help setting up the study and obtain ethics approval. MA, CS, BV were responsible for recruiting subjects for the study. MC, LV, MK, GM contributed to assessment of subjects and data analysis. JS, JP, PFP all contributed to the recruitment of subjects and set up of the study. PP and PFP critically revised the manuscript. OH, PA, PMG obtained the grant to set up the study and supervised the whole work and critically revised the manuscript. PFP and PMG substantially contributed to the ideation of the study.

All authors revised the manuscript, working together towards its final completion.

## Funding

This study was funded by Medical Research Council-UK (no. MC-A656-5QD30), Maudsley Charity (no. 666), Brain and Behavior Research Foundation, and Wellcome Trust (no. 094849/Z/10/Z) grants to HO and the National Institute for Health Research (NIHR) Biomedical Research Centre at South London and Maudsley NHS Foundation Trust and King’s College London. MK was funded by a Medical Research Council Fellowship (grant MR/J008915/1). We would like to acknowledge the support of The European Network of National Networks studying Gene-Environment Interactions in Schizophrenia” (EU-GEI), which is supported by funding from the European Union [European Community’s Seventh Framework Program (HEALTH-F2-2009–241909; Project EU-GEI)]. MB was supported by a Rubicon grant from the Netherlands Organisation for Scientific Research (NWO 825.11.034). ST is supported by a Brain and Behavior Young Investigator award (NARSAD YI, 24786) and by a Maudsley Charity Grant (2018/16).

GM is supported by a Sir Henry Dale Fellowship, jointly funded by the Wellcome Trust and the Royal Society (#202397/Z/16/Z).

MG was supported by a Veni fellowship from the Netherlands Organisation for Scientific Research (grant number 016.166.038)

## Conflicts of Interest Statement

OH has received investigator-initiated research funding from and/or participated in advisory/speaker meetings organized by Astra-Zeneca, Autifony, BMS, Eli Lilly, Heptares, Jansenn, Lundbeck, Lyden-Delta, Otsuka, Servier, Sunovion, Rand, and Roche. PF has received research funding and/or participated in advisory/speaker meetings organized by Lundbeck. Neither OH nor his family have been employed by or have holdings/a financial stake in any biomedical company.

The remaining authors declare that the research was conducted in the absence of any commercial or financial relationships that could be construed as a potential conflict of interest.
